# The five dimensions of receptor pharmacology exemplified by melatonin receptors: An opinion

**DOI:** 10.1002/prp2.556

**Published:** 2019-12-29

**Authors:** Jean A. Boutin, Céline Legros

**Affiliations:** ^1^ Institut de Recherches Internationales Servier Suresnes France; ^2^ Institut de Recherches Servier Croissy‐sur‐Seine France

**Keywords:** antagonism, biased ligands, dimerization, expression, GPCRs, interactome, melatonin, review

## Abstract

Receptology has been complicated with enhancements in our knowledge of G‐protein‐coupled‐receptor (GPCR) biochemistry. This complexity is exemplified by the pharmacology of melatonin receptors. Here, we describe the complexity of GPCR biochemistry in five dimensions: (a) receptor expression, particularly in organs/tissues that are only partially understood; (b) ligands and receptor‐associated proteins (interactome); (c) receptor function, which might be more complex than the known G‐protein‐coupled systems; (d) ligand bias, which favors a particular pathway; and (e) receptor dimerization, which might concern all receptors coexpressed in the same cell. Thus, receptor signaling might be modified or modulated, depending on the nature of the receptor complex. Fundamental studies are needed to clarify these points and find new ways to tackle receptor functionality. This opinion article emphasizes the global questions attached to new descriptions of GPCRs and aims to raise our awareness of the tremendous complexity of modern receptology.

**Abbreviations**
BRET: Bioluminescence Resonance Energy Transfer
FRET: Förster (or Fluorescence) resonance energy transfer
GPCR: G‐protein‐coupled‐receptor
MRR: melatonin‐related receptor

## 1 RECEPTORS AND DRUGS: THE PAST

Until fairly recently, new ligands for receptors were frequently discovered, because most drugs in the Pharmacopeia were receptor antagonists.^1^ Receptors are integral membrane proteins that transduce signals from the outside to inside cells. Receptor ligands are highly variable in nature, ranging from small molecules and photons to peptides and small proteins. Once the ligand is attached to the cognate receptor, a signal is transduced via intracellular machinery. When this machinery was mainly linked to G‐proteins,^2^ the receptor is called a G‐protein‐coupled receptor (GPCR). However, current studies have shown that GPCRs transduce signals via pathways outside the canonical G‐protein pathway.^2^ GPCRs comprise a family of structurally, closely related transmembrane proteins. Their structures are nearly identical: a N‐terminus is located outside the cell, and seven transmembrane domains are separated by three external domains—or loops—and three internal loops. The receptor's C‐terminus is located inside the cell. Different receptors have different sized loops and N‐ and C‐termini. The general structure of GPCRs was described by crystallographers and structuralists^3^, ^4^, ^5^; all GPCRs have a quite uniform structure, including melatonin receptors.^6^, ^7^ Indeed, the field of melatonin receptors has made remarkably important structural advances, as recently reviewed by Cecon et al.^8^

For the time being, receptors remain the main targets for drugs. In addition, they are an important source of new research on future drugs.^9^

## 2 RECEPTORS AND DRUGS, THE PRESENT

Currently, the picture has become occluded, but scientifically more exciting. The almost 2D space explored with GPCR research (ligand & functionality) in the 1980s has become a 5D space today. Current GCPR research focuses on five characteristics expression, ligands/associated proteins, functionality, biases, and dimerization.

The first dimension is expression. It is important to determine, in a given native cellular condition (as opposed to recombinant receptor‐expressing cellular conditions), whether the receptors that can heterodimerize are actually expressed in the same cells at the same time.

Second, it was discovered that receptors have many binding pockets, apart from their canonical agonist‐/antagonist‐binding sites. This discovery led to a categorization of ligands. Some are neutral allosteric modulators, and others are negative or positive allosteric modulators. Furthermore, agonists and antagonists have become subcategorized as full, partial, or inverse activators, based on how their binding affects receptor activity. GCPR studies have also shown that the receptor protein is coupled to G‐proteins, but also to hundreds of proteins (interactome) the influence of which on binding and functions remains unknown.

Third, receptor functions depend on the nature of ligand but also on the signaling routes that the binding of the ligand induces. However, the relationships between the interactome proteins and the receptor remain unclear, beyond the classical G‐protein signaling pathways. These interactome proteins might change the affinity of the ligand(s) for the receptor; they might modulate the functionality of the receptor upon ligand binding; or they might confer a new function that was previously unknown.

Fourth, the term “biased” ligands was coined^10^, ^11^, ^12^, ^13^ to describe ligand compounds with different functionalities. These ligands can lead to different downstream pathways, depending on their bound structure.^14^ Therefore, it has become increasingly complex to describe, for example, the profile of a given antagonist. One must test the different functionalities of the receptor under different conditions in the presence of the given compound to establish the rules for describing a precise biased profile.

Finally, fifth, it remains to be established whether any given GPCR subunit might be capable of both homodimerization and heterodimerization. For example, under some conditions, receptor A might be able to homodimerize to form receptor AA, with specific properties. However, under other conditions, receptor A might heterodimerize with receptor B to form a stable receptor AB complex, with some characteristics of A and some characteristics of B; thus, its functional characteristics are different from those of the respective homodimers.^15^, ^16^ In some cases, it has been shown that the functions of a heterodimer were not the same as those of the homodimer. However, it remains unclear whether any GPCR is capable of heterodimerizing with any other GPCR. This scenario would be a nightmare for pharmacologists and drug developers. Furthermore, it is not clear how to link the functionality of a receptor or heterocomplex to a particular pathology.

To illustrate some of these points, we will briefly describe what is known, roughly, for our preferred receptors: melatonin receptors. Melatonin pharmacology is special, due to the high affinity of melatonin for its receptors (Ki ≤ 1 nmol L^−1^) and due to the current search for agonists. Moreover, we believe that the problems linked to melatonin receptor pharmacology are representative of current problems in receptology.

## 3 THE 5D SPACE, EXEMPLIFIED BY MELATONIN RECEPTORS

Melatonin is a neurohormone with many different features.^17^, ^18^, ^19^, ^20^, ^21^, ^22^, ^23^ To our knowledge, it acts through two main melatonin receptor subtypes, MT_1_ and MT_2_, but the list of proteins targeted by melatonin has grown over the years.^24^ MT_1_ and MT_2_ have shown almost no difference in pharmacology. Despite the synthesis of several hundreds of agonist ligands, only a handful are specific for MT_2_ over MT_1_, and only a single ligand^25^ is specific for MT_1_ over MT_2_. Several excellent reviews have detailed the pharmacology of these receptors.^26^, ^27^, ^28^ Most studies on melatonin receptor pharmacology were conducted with recombinant receptors expressed in orthologous cells. Only a small set of data has been acquired with native tissues (see Table 1). Once a receptor is expressed, there is a limited panel of parameters to consider; here, we describe the five key parameters.

**Table 1:** Melatonin‐regulated signaling responses in cells or tissues with endogenous receptors

Signaling response	Cells/tissues	Functional effect	Predominant receptor subtype^a^	References
cAMP formation	**Ovine *pars tuberalis* primary cells**	↓	MT_1_	McNulty et al^48^
	**Rat neonatal pituitary primary cells**	↓	MT_1_/MT_2_	Vanecek and Vollrath^49^
				Slanar et al^46^
	SCN2.2 cells	↓	MT_1_/MT_2_	Rivera‐Bermúdez et al^50^
pCREB	**Mouse SCN slices**	↓	MT_1_	von Gall et al^51^
	**Ovine *pars tuberalis* primary cells**	↓	MT_1_	McNulty et al^48^
pJNK, pERK1/2	MCF‐7 cells	↑	MT_1_	Chan et al^52^
PKC activity	**Rat SCN slices**	↑	MT_2_	Hunt et al^53^
	SCN2.2 cells	↑	MT_2_	Gerdin et al^54^
				Rivera‐Bermúdez et al^50^
	**Prostate epithelial cells**	↓	MT_1_/MT_2_	Gilad et al^55^
BKCa: large‐conductance Ca^2+^‐dependent K^+^ channels	**Rat cerebral arteries**	↓	MT_1_/MT_2_	Geary et al^44^
	**Rat tail arteries**	↓	MT_1_/MT_2_	Geary et al^45^
K^+^ conductance	**Mouse SCN slices**	↑	MT_1_/MT_2_	Jiang et al^42^
Inward‐rectifying cation current (*I_k_*)	**Mouse SCN slices**	↓	MT_1_/ MT_2_	Jiang et al^42^
GABA_A_‐mediated current	**Rat SCN slices**	↑	MT_1_	Wan et al^47^
	**Rat hippocampus slices**	↓	MT_2_	Wan et al^47^
Ca^2+^ influx	**Rat neonatal pituitary primary cells**	↓	MT_1_/ MT_2_	Slanar et al^46^
Intracellular Ca^2+^	**Ovine *pars tuberalis* primary cells**	↑	MT_1_	Brydon et al^43^

Note

↓: a decrease or inhibition; ↑: an increase or activation.

Abbreviation: SCN, suprachiasmatic nucleus.

a Reported after the use of antagonist‐based pharmacological experiments. Natural material sources (as opposed to recombinant sources) (in bold).

### 3.1 Expression (and coexpression)

Intuitively, it is clear that receptors can be active only when they are expressed in a given organ/cell. Similarly, receptors can dimerize (see section 3.5) only when both element of the dimer are expressed in the same cell at the same time. However, the cellular expression of receptors has not been mapped as closely as might be imagined. Most expression patterns were identified with quantitative PCR (qPCR). Therefore, only receptor mRNA expression levels are known with some accuracy. The main problem impeding mapping is the lack of specific antibodies. Monoclonal antibodies should be preferred over polyclonal ones. For the melatonin receptors, productions have been unsuccessfully attempted for at least two decades. It is only in the last few months, that a publication described two monoclonal antibodies; one specific for each melatonin receptor subtype.^29^ This was a major step toward the quantification of melatonin receptors in various tissues, particularly ex vivo. Furthermore, the new antibodies were shown to be efficient and specific in Western blots, immunoprecipitation, immunofluorescence, and a proximity ligation assay; in other words, these antibodies represent the perfect instrument for localizing melatonin receptor subtype expression.

Furthermore, our colleagues from Lincoln University also developed new antibodies that detected melatonin receptors in some parts of the brain, but not other parts, which led to a breakthrough in the field. These new tools will finally make it possible to address multiple questions, including heterodimerization, and they might simplify the currently complex picture of GCPRs (Mosely & Ngomba, personal communications).

### 3.2 Binding and receptor‐associated molecules: ligands and the interactome

**Figure 1:**
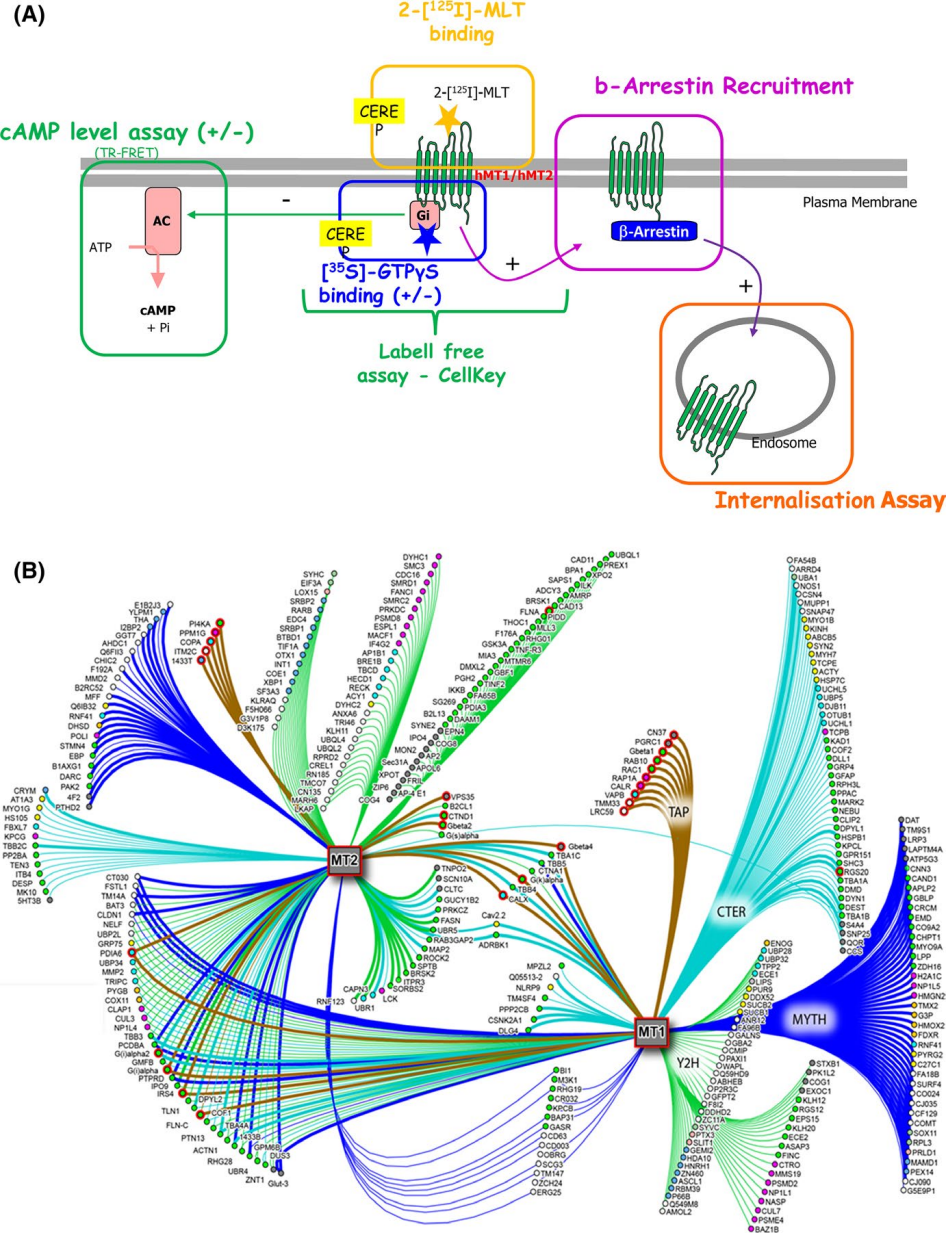
Receptor functions: the functionality of a receptor depends on the associated proteins. (A) The classic downstream pathways (reproduced from the accompanying publication Legros et al^57^). These main signaling pathways were discovered in classical receptor studies. (B) A summary of the results of double hybrid experiments. Approximately 350 proteins were experimentally found to be associated with MT_1_ (*left*) or MT_2_ (*right*) receptors (reproduced with permission from Benleulmi‐Chaachoua et al,^39^). The original figure legend applies here: “Melatonin receptor network, based on 20 different screens. Identified proteins are clustered based on the detection method used. Edge colors identify the identification methods applied as defined: dark blue for the MYTH, blue for Cter peptide purification, green for the Y2H, and brown for the TAP. Thick lines correspond to confirmed protein‐protein interactions (PPIs) and node colors refer to Gene Ontology (GO) biological function. The network contains 366 interacting partners of which 168, 143, and 52 are specific for MT_1_, MT_2_, or common for both receptors, respectively. Additional interactions and partners were added from the Interologou Interaction Database I2D Ver. 2.3. The network is visualized using NAViGaTOR 2.3.1 tools (http://ophid.utoronto.ca/navigator). Classifications of PPIs of the MT_1_ and MT_2_ receptor interactomes depend on GO entries”

Receptors can be characterized according to two functions: ligand binding on the outside of the plasma membrane and interactome activity inside the cell, typically in the vicinity of the C‐terminus. The ligand could be considered the messenger, and the interactome could be considered the message transmitter. Natural ligands are often agonists. Most agonists are not impeded by a natural antagonist—until we'll find counter examples, such as GPCR peptide ligands that are believed to be antagonized by shorter versions of the same peptides, but most of the time, natural small molecule antagonists remain elusive (and thus, unknown). There is a large difference between our current view of melatonin receptors, which includes the activation of rather standard pathways, and the universe of proteins produced from double hybrid experiments (see Figure 1).

#### 3.2.1 Ligand binding

Briefly, melatonin is the natural ligand of MT_1_ and MT_2_. Melatonin is synthesized in the brain, and it travels freely through membranes. Its presence in blood displays a circadian rhythmicity: it is low during the day (5 pg ml^−1^) and high at night (100‐150 pg ml^−1^), although there are variations between individuals and changes occur over the course of a lifetime (concentrations decline with age). The circadian rhythm regulates both the receptor^30^ and the synthesis of melatonin.^31^ Therefore, one of the first ways to regulate melatonin receptor activity was the availability of the natural ligand. The ingenuity of researchers has given us an analogue of natural melatonin, 2‐[^125^I]‐melatonin,^32^ which can be used to measure the affinity of the ligand for its receptor. Indeed, combined with a radiotracer, such as the traditional 2‐[^125^I]‐melatonin,^32^ or more recently, the triple‐tritiated melatonin,^33^ hundreds of melatonin‐like compounds have been described for their capacity to bind to melatonin receptors (see a chemical review of those molecules in Zlotos et al^34^). More recent studies have given rise to a new generation of compounds that are more selective for one of the MT_1_ or MT_2_ subtypes.^35^ Interestingly, melatonin receptors are slightly different from other receptors, because we were essentially only seeking agonists, as opposed to the traditional search for antagonists as drugs for most of the other GPCRs. Currently, melatonin receptor antagonists are mostly unavailable, particularly when one wants to use them in vivo. This peculiarity might be explained by the fact that antagonist compounds have not been considered in a therapeutic context. A “side effect” of ligand chemistry is that it can produce new types of compounds, such as nonpenetrating molecules,^36^ fluorescent ligands,^37^ and new radiolabeled ligands,^33^, ^38^ which have contributed to a new understanding of melatonin receptor pharmacology. On the other hand, the production of many compounds that are only slightly different from one another should, in the long run, extend our understanding of the relationship between the structure of a ligand, its topological pause in the receptor‐binding site,^8^ and its bias, in terms of receptor functionality (see sections 3.3 & 3.4).

#### 3.2.2 The interactome

The interactome is the ensemble of proteins suspected to interact with a protein, for example, a receptor. However, proteins rarely act on their own in cellular context. In a seminal review, Jockers and coworkers^39^ comprised a list of the proteins that interacted with melatonin receptors, at either the N‐ or C‐terminal domains. They found that about 350 different gene products could interact with these receptors (see Figure 1B). Of course, this list simply comprised a catalog, although an important catalogue. For most of those proteins, it remains unknown how they influence melatonin pathway signaling. This lack of knowledge led us to speculate that the mechanism underlying signal transmission inside the cell could be far more complex than the current simplified notion that it is mostly driven by G‐proteins. It will take years for us to complement those findings with independent observations on how those—or some of those—proteins influence the binding or functionality of the melatonin receptors.

### 3.3 Receptor function: the G‐protein coupling processes

G‐proteins are extremely numerous. Their expression is regulated at the gene level and they might also be regulated at the translation level. Furthermore, their ability to couple with a given receptor requires the coexpression of the receptor. However, due to the limited access to pharmacology on ex vivo tissues that naturally express melatonin receptor(s), researchers generally do not double‐check whether the actual G‐proteins of interest are expressed. Moreover, despite the many studies on G‐proteins, their coexpression in native tissues remains poorly documented. Furthermore, the roles of G‐proteins are largely simplified (see Figure 1A) and probably underestimated. For example, when the Gβγ complex is bound to a histamine receptor, it can activate phospholipase A2^40^; but when the same complex is bound to a muscarinic acetylcholine receptor, it directly leads to the opening of G‐protein‐coupled inward‐rectifying potassium channels.^41^ The Gβγ complex can also activate L‐type calcium channels, as in H_3_ receptor pharmacology. Thus, the simple picture that, for decades, described receptor functionality has now become only a small part of the picture.

As mentioned earlier, most studies on melatonin receptors were performed with recombinant systems. The exceptions are quite rare; Table 1 shows unconventional melatonin signaling pathways, which suggest that the view we have of receptor signaling is probably only a partial picture. Undoubtedly, this observation is likely to extend to our views of all GPCRs. For melatonin receptors, Table 1 shows data from both ex vivo and in cellulo experiments (see also complete review by Cecon et al, 2018)^27^ from untransformed systems (as opposed to transfected cells). In the table, several studies by different groups showed the interactions between melatonin, the melatonin receptor, and an ion conductance,^42^ including calcium movements,^43^, ^44^, ^45^, ^46^ observed in different models. Furthermore, some studies showed coupling between melatonin receptors and GABA receptors,^47^ which initiated a new line of research between the neurohormone and this key neuronal process. Importantly, these experiments were conducted in untransformed ex vivo models (highlighted in yellow in Table 1).

We added to this picture when we determined new signaling routes downstream of melatonin receptors.^56^ Our findings might facilitate deciphering biased ligand behaviors (see below).^57^ Furthermore, as shown by others, the MT_1_ receptor was associated with the Mupp1 protein,^58^ which regulates receptor coupling with the Gi‐protein. As observed in the interactome studies, the receptors were sometimes coupled with unexpected pathways/proteins. Considering the number of “new” proteins identified in the interactome experiments, it would be surprising if alternative pathways were not discovered, unless the interactome approach (mostly double hybrid experiments^59^) resulted in a large number of false positives, as previously discussed.^60^ However, apart from potential false positives, many of the proteins identified are likely to influence the expression, the half‐life, or the coupling of melatonin receptors. The experiments shown in Table 1 are essential, because they have opened up an avenue of research that could lead to a better understanding of the signaling context. Nevertheless, the identification of proteins is insufficient, by far, for addressing questions like: how does this particular protein change the overall behavior of the receptor and/or the natural agonist under investigation? The functions of some proteins are known, such as G proteins or proteins that form the ubiquitinome (and thus, regulate the target receptor). However, in most cases, this question must be addressed with biological experiments in a wet lab.

### 3.4 Receptor function: the bias of the ligand

**Figure 2:**
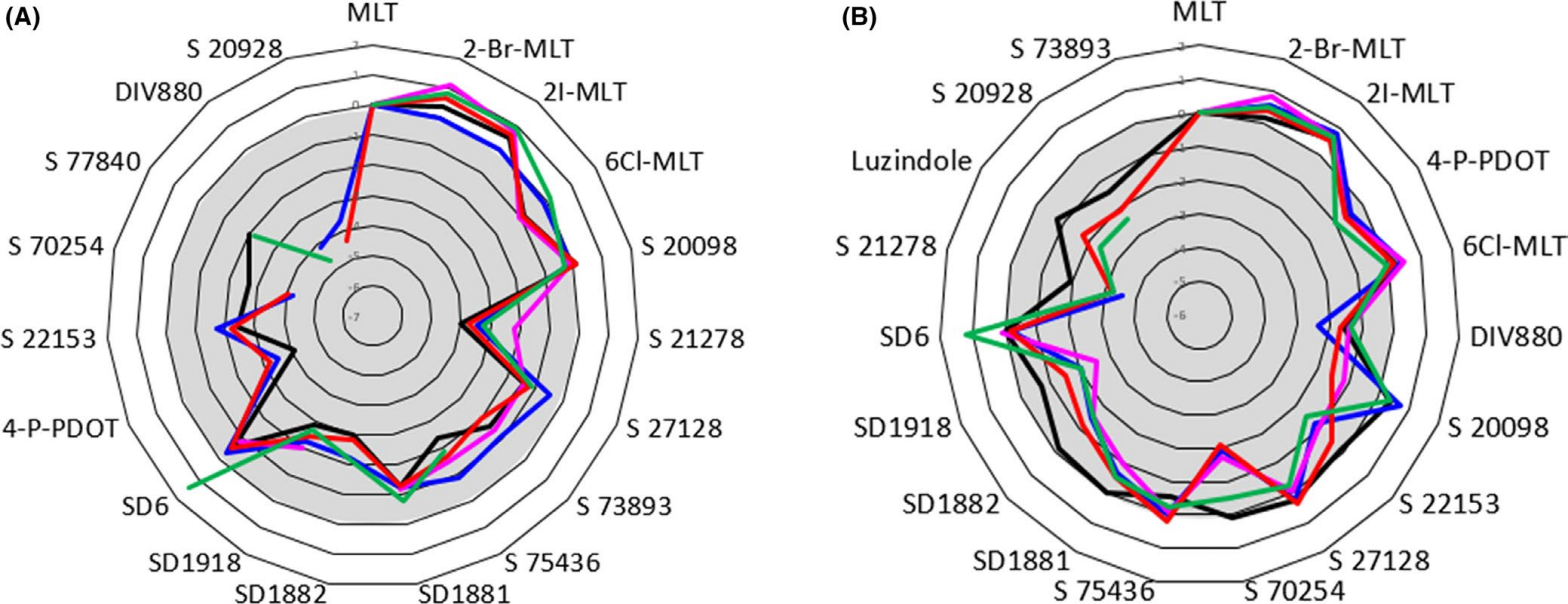
Biased ligand radar‐plot representations of melatonin receptor agonists. Data represent ΔLog(max/EC50) values, typically relevant from biased pharmacology^57^ which compare the agonism of the compound to the agonism of melatonin for the particular signaling pathway. The set of ligands comprised 19 ligands, including melatonin. (A) MT_1_ receptor; (B) MT_2_ receptor. The traces are color‐coded as follows: green = GTPγS; blue = β‐arrestin; pink = internalization; red = cAMP; and black = Cellkey^®^ (reproduced from the accompanying publication^57^)

Activation of GPCRs by agonists rarely lead to the activation of all signaling pathways mediated by a given receptor. There are biased agonists produce subsets of receptor behaviors compared to other ligands. This functional selectivity is cell‐type dependent.^10^ The main goal of melatonin research is to obtain functional analogues of melatonin. Consequently, most of the compounds synthesized over the last three decades were agonists. For the record, about 2000 melatonin analogues have been published, and very little research has been conducted to describe antagonists (JA Boutin, P Witt‐Enderby, D Zlotos, in preparation). To the best of our knowledge, our previous study^57^ was the first to address a large number of compounds in many different GPCR‐signaling pathways, even when considering studies outside the melatonin field. That accompanying paper^57^ showed that some compounds—probably five—acted as antagonists to at least one of the melatonin receptor subtypes. Nevertheless, the specificity of those compounds, which is useful to know for in vivo experiments, was far from convincing. Further exploration of the characteristics of those antagonists will be necessary to generate new, more potent antagonists. Based on our screening of the classical melatonin ligands,^57^ the effect sizes of biased ligands were modest, but there were critical differences between agonist functionalities (see Figure 2).

Despite the relatively low diversity of melatonin receptor ligands, it is essential to conduct structure‐activity relationship experiments on biased ligands. Spadoni et al^61^ described a common motif among the many agonists that have been synthesized and described: they all bear a unique bicyclic aromatic structure bearing an aliphatic chain with a amide function, barely mimicking the melatonin structure. Those basic structures were decorated with a paramount of functions (see Zlotos et al^34^ and Spadoni et al^61^ for chemical details). Moreover, the crystal structures of MT_1_ and MT_2_ receptors were recently published.^6^, ^7^ These structures provide the opportunity to find more diverse antagonistic compounds. However, because the structures crystallized were inactive receptors in their low affinity state, the ab initio discovery of new ligands will probably be delayed. Structure‐function information about ligands and receptors will facilitate predictions of the biased nature of new ligands, in light of functionalities that we can measure.^62^, ^63^

### 3.5 Homo‐ and heterodimerization

As reviewed by Ferré et al,^64^ GPCRs are known to form oligomers, either with their own species (eg, serotonin homodimers) or with other, similar species (eg, a serotonin receptor with an adrenergic receptor to form a heterodimeric complex).^63^, ^65^, ^66^ Paramount examples can be viewed at a specialized website (http://data.gpcr-okb.org/gpcr-okb/), see also Nemoto et al^67^ for further information on oligomerization of receptors. Evidence of dimerization can be found with several types of experiments,^64^ including Förster (or Fluorescence) resonance energy transfer (FRET), Bioluminescence resonance energy transfer (BRET), and receptor crystallography.^68^ It has also been shown, in some instances, that heterodimerization provided a role for orphan receptors.^69^, ^70^ Indeed, orphan receptors are integral membrane proteins with structures that undoubtedly belong to the GPCR family, but they have no known ligand. In the melatonin receptor family, an orphan receptor has been identified; it is known as GPR50 (previously known as melatonin‐related receptor, MRR). Despite its family resemblance, it lacks any capacity to bind melatonin in mammals (except in platypus),^71^ and it is closely related to Mel1c, the third melatonin receptor found in batrachians and birds.^71^, ^72^ However, it remains unknown whether GPCR heterodimers are physiologically relevant.^73^

Understanding homodimerization has made it possible to synthesize and characterize homodimeric ligands^74^, ^75^ that can act as potent antagonists, as previously described for melatonin receptors. These ligands showed a slightly enhanced selectivity for the MT_1_ subtype.^75^

Although receptor homodimerization does not appear to increase the complexity of receptor pharmacology, heterodimerization is completely different. As pointed out in a review by Kamal & Jockers,^76^ heterodimerization might have an important impact on neuroendocrinology and on all parts of receptology that are applied to therapeutics. It was established that melatonin receptors could heterodimerize, either with the other subtype (ie, MT_1_ with MT_2_)^63^ or with GPR50.^66^ In heterodimeric complexes, the receptor function can shift from pure melatonin signal transduction to a mixture of transduction pathways (eg, melatonin and serotonin pathways) or it can be altered by GPR50 heterodimerization^66^ (see discussion in Jockers et al^77^). The effects of heterodimerization are more dramatic when the two receptors do not belong to the same family (eg, they do not recognize the same ligand/type of ligand). There are endless potential modifications that might occur in the signaling pathway of one receptor by activating the pathway of another receptor. Indeed, receptor heterodimerization might explain the antidepressive profile of Agomelatin^®^; that is, the effect might only occur when it acts on a melatonin/5HT2c serotonin heterocomplex.^78^

The worry associated with heterodimerization is that, if both receptors are expressed in the same cells and they are present in close vicinity in the membrane, then they probably will dimerize, which could change the way the agonist induces intracellular signaling. Thus, ligand pharmacology should be revisited, with the hypothesis that its “main” receptor target can dimerize with other receptors present in the same cells. It might be somewhat easier to study these systems with purified receptors that maintain their signaling pathway(s) and a specialized approach, as described for the ghrelin receptor.^79^ Furthermore, despite many difficulties, essentially due to poor MT_1_ stability, we obtained pure receptors in the active form, and they could couple^25^ with a G‐protein. Although, we are gaining quite a lot of knowledge about the concept of dimerization, the ability to predict appears to remain far in the future.^80^

## 4 CONCLUSIVE REMARKS

Interestingly, the more layers of knowledge we gain about the way receptors function, the more we are surprised that the receptor antagonists discovered long before we gained this knowledge were excellent drugs for treating pathological problems, particularly in the neurosciences. As we investigated ideas for this study, it occurred to us that the classic agonist/antagonist concept had potentially passed its usefulness. In fact, this concept might not have evolved in vivo (wishful thinking?). Instead, the different functions we observe with different ligands might be due to (GPCR‐GPCR) heterodimerization. Alternatively, the different observed functions could be due to the participation of adjuvant proteins that regulate the downstream pathways and ligand binding by locally changing the availability of different parts of the receptor(s). This mechanism currently remains beyond our complete understanding. However, it was shown for calcium regulators. For example, the binding of ligand(s) to the ryanodine receptor (1 MDa) was regulated by extremely small proteins (12.5 kDa), which locally regulated the functionality of the receptor.^81^

This area might be interesting to explore, because it could broaden our understanding—and predictions—of ligand binding and how they gain access to the receptor‐binding site. Currently, fantastic progress has been made with both crystallography and cryoelectronic microscopy,^82^ based on previously obtained data. Taken together with the large body of knowledge about the surface of GPCRs, thanks to the pioneers in receptor crystallography,^83^, ^84^ we have no doubt that the new decade will see clear progress in the prediction of biased ligand behaviors.

The next evolution, which we have attempted, very modestly, to introduce into our daily work, is to build cellular models relevant to a given pathology. The recent surge in stem cell biology and use has opened up immense possibilities.^85^ First, in integrated receptology, with stem cells, we could develop cells that closely resemble “normal” cells (as opposed to cells manipulated in laboratories, which are often cancer cell‐derived). With this approach, we might develop actual in situ receptor biochemistry, where the receptor is expressed in the presence of its necessary protein machinery and context; thus, we could collect data closer to the “real life” situation. Second, by introducing point mutation(s) in receptor sequences that are relevant to the overall pathology, perhaps based on the systematic genotyping of diseases, we might be able to study the effects of a series of ligands more closely, taking into consideration the global, five‐dimensional space described here.

Again, these concepts are relevant to studies both within and outside the melatonin field.

## DISCLOSURE

The authors declare no conflict of interest.

## AUTHOR CONTRIBUTIONS

Both authors were involved in the writing of the review.
